# Combination of Metabolomic Analysis and Transcriptomic Analysis Reveals Differential Mechanism of Phenylpropanoid Biosynthesis and Flavonoid Biosynthesis in Wild and Cultivated Forms of *Angelica sinensis*

**DOI:** 10.3390/metabo15090633

**Published:** 2025-09-22

**Authors:** Yuanyuan Wang, Jialing Zhang, Yiyang Chen, Juanjuan Liu, Ke Li, Ling Jin

**Affiliations:** 1 School of Pharmacy, Gansu University of Traditional Chinese Medicine, Lanzhou 730101, China; gswyy@gszy.edu.cn (Y.W.); zhjl3615@163.com (J.Z.); 18418656695@163.com (K.L.); 2 School of Pharmacy, Nanjing University of Traditional Chinese Medicine, Nanjing 210023, China; chenyy001012@163.com; 3 Resource Center for Chinese Materia Medica, Institute of Chinese Materia Medica, China Academy of Chinese Medical Sciences, Beijing 100700, China; gszyljj2023@163.com; 4 Gansu Pharmaceutical Industry Innovation Research Institute, Lanzhou 730000, China; 5 Northwest Collaborative Innovation Center for Traditional Chinese Medicine Co-Constructed by Gansu Province & MOE of PRC, Lanzhou 730000, China

**Keywords:** *Angelica sinensis*, wild, cultivated, phenylpropanoid biosynthesis, flavonoid biosynthesis

## Abstract

Objectives: *Angelica sinensis* is a type of traditional Chinese medicine (TCM) used primarily as a blood tonic. The chemical components that exert their efficacy are mainly bioactive metabolites, such as ferulic acid, flavonoids, and volatile oils. The resources of wild *Angelica sinensis* (WA) are very scarce, and almost all the market circulation of TCM formulations relies on cultivated *Angelica sinensis* (CA). Some studies have shown that WA and CA differ in morphological features and chemical composition, but the reasons and mechanisms behind the differences have not been studied deeply. Methods: Herein, metabolomics analysis (MA) and transcriptomics analysis (TA) were used to reveal the differences in bioactive metabolites and genes between WA and CA. Expression of key genes was verified by real-time reverse transcription-quantitative polymerase chain reaction (RT-qPCR). Results: Results showed that 12,580 differential metabolites (DMs) and 1837 differentially expressed genes (DEGs) were identified between WA and CA. Fourteen DMs (e.g., cinnamic acid, caffeic acid, ferulic acid, *p*-coumaroylquinic acid, and phlorizin) and 27 DEGs (e.g., cinnamic acid 4-hydroxylase (*C4H*), 4-coumarate-CoA ligase (*4CL*), shikimate O-hydroxycinnamoyltransferase (*HCT*), caffeic acid-O-methyltransferase (*COMT*), cinnamyl-alcohol dehydrogenase (*CAD*), flavonol synthase (*FLS*)) were screened in phenylpropanoid biosynthesis and flavonoid biosynthesis. A combined analysis of MA and TA was performed, and a network map of DMs regulated by DEGs was plotted. The results of real-time RT-qPCR showed that the transcriptome data were reliable. Conclusions: These findings provide a reference for further optimization of the development of WA cultivation and breeding of CA varieties.

## 1. Introduction

*Angelicae sinensis* Radix is the dried root of *Angelica sinensis* (Oliv.) Diels of the Apiaceae family. If administered, it can replenish and activate blood, regulate menstruation to relieve pain, and clean the intestines to relax the bowels. It is a type of traditional Chinese medicine (TCM) used primarily for the syndrome of blood deficiency and blood stasis [1].

Studies have shown that the resources of wild *Angelica sinensis* (WA) are limited to some alpine regions and unfrequented mountain jungles in Gansu, Sichuan, Tibet, Yunnan, and other provinces in China. These natural habitats are characterized by extreme and uncontrollable selective pressures, including severe cold, nutrient-poor soils, natural shading, and intense interspecific competition. Under such conditions, WA exhibits slow growth, irregular morphology, and a higher accumulation of secondary metabolites [2]. Due to the increase in demand for *A. sinensis* and the increase in prices, the resources of WA have decreased sharply. In contrast, cultivated *A. sinensis* (CA) is grown under human-managed agricultural conditions, with optimized soil fertility, regulated irrigation, controlled light exposure, and systematic pest and disease management, effectively eliminating most environmental stresses. As a result, almost all of the *A. sinensis* on the market is cultivated [3].

Some studies have shown that WA and CA differ in morphological features and chemical composition. WA is small, with dark purple-brown skin and a strong fragrance, whereas CA is larger, with light purple-brown skin and a light fragrance [4]. The content of organic acids, volatile oils, and polysaccharides differs between WA and CA [5]. These chemical components exert cardiovascular, cerebrovascular, hepatoprotective, anti-inflammatory, and anti-tumor effects [1,6,7,8,9]. Systematic research on the differences between WA and CA is lacking. Most studies have focused on a comparative analysis of the morphological features and chemical composition of WA and CA. Comparative studies at the molecular level are lacking.

“Omics” technologies, such as transcriptomics analysis (TA) and metabolomics analysis (MA), can explain the complexity of molecules at multiple levels. The complex composition, complex mechanism of action, and target diversity of traditional Chinese medicine formulations (TCMFs) indicate that their chemical composition must be analyzed from multiple approaches and aspects, and this can be achieved by combining omics technologies [10]. The combination of several omics technologies and the integration of different omics methods have significant advantages for the study of TCMFs [11].

The causes and mechanisms of the differences in morphological features and chemical composition between WA and CA have not been studied in depth. We analyzed the differences in metabolites and key genes between WA and CA based on MA and TA. Our data could be a foundation for the study of the differences in morphological features and chemical composition between WA and CA at the molecular level. Such investigations could aid exploration of the alternative use of WA and CA.

## 2. Materials and Methods

### 2.1. Plant Material

**Figure 1:**
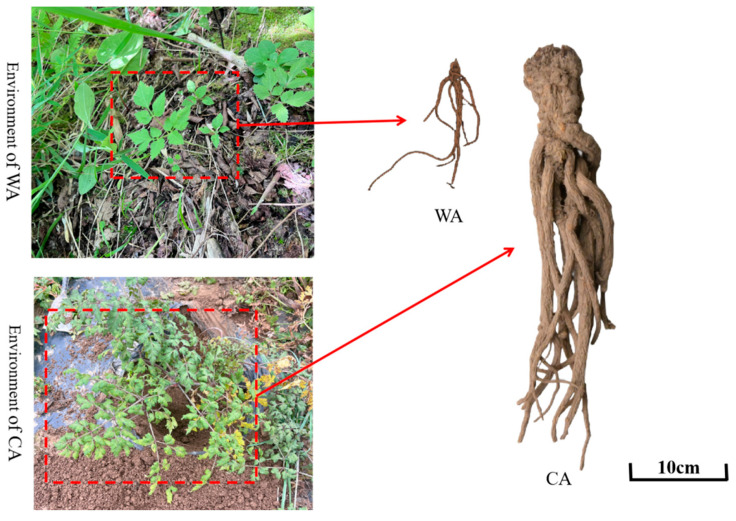
Environment and morphological features of WA and CA.

The first year of *A. sinensis* is the seedling period, the second year is to harvest the fleshy roots, which are to be used as TCM, and the third year is to bolt and bloom for harvesting seeds [12]. The bolting makes the fleshy roots of *A. sinensis* lignified, which cannot be used as TCM. Therefore, the growth age of the *A. sinensis* samples selected in this study was two years. The fresh roots of WA (WA-1, WA-2, WA-3) and CA (CA-1, CA-2, CA-3) were collected as experimental materials (Figure 1). Samples were collected from Nanhe Town (2113 m above sea level; 104°18′50.27″ E, 34°08′57.71″ N) in Tanchang County (Dingxi City, Gansu Province, China) in September 2023. They were identified by Professor Jin Ling (Gansu University of Traditional Chinese Medicine, Lanzhou, China). Collected samples were scrubbed and transported, frozen immediately in liquid nitrogen, and stored at −80 °C for MA and TA.

### 2.2. Metabolomics Analysis

#### 2.2.1. Extraction of Metabolites

Freeze-dried samples were ground. Each sample (20 mg) was weighed in an Eppendorf™ tube. Then, 1 mL of an extract solution (methanol–acetonitrile–water = 2:2:1, with an isotopically labeled internal standard mixture) was added. Next, samples were homogenized (35 Hz, 4 min) and then sonicated for 5 min in an ice–water bath. This cycle of homogenization and sonication was repeated three times. Then, samples were incubated for 1 h at −40 °C and centrifuged (13,800× *g* for 15 min at 4 °C). The supernatant was filtered carefully through a 0.22 μm microporous membrane and transferred to a fresh glass vial for analysis.

#### 2.2.2. Liquid Chromatography–Tandem Mass Spectrometry (LC-MS/MS)

LC-MS/MS was performed using an ultrahigh performance liquid chromatography (UHPLC) system (Vanquish; Thermo Fisher Scientific, Waltham, MA, USA) with a Phenomenex Kinetex C18 (2.1 mm × 100 mm, 2.6 μm) column coupled to a mass spectrometer (Orbitrap Exploris 120; Thermo Fisher Scientific).

The mobile phase consisted of 0.01% acetic acid in water (A) and isopropanol–acetonitrile (1:1, *v*/*v*) (B). The auto-sampler was maintained at a temperature of 4 °C, and the injection volume was set to 2 μL. MS/MS spectra were obtained using a mass spectrometer operating in information-dependent acquisition mode via the Xcalibu software, version 4.2 (Thermo Fisher Scientific). This mode allows the software to continuously assess the full-scan MS spectrum. The electrospray ionization source parameters were configured as follows: sheath gas flow rate was 50 Arb; auxiliary gas flow rate at 15 Arb; capillary temperature at 320 °C; full MS resolution at 60,000; MS/MS resolution at 15,000; and collision energy set at 20/30/40 in Single-Nanoparticle Collision Electrochemistry (SNCE) mode. The spray voltage was adjusted to 3.8 kV for positive ion mode and −3.4 kV for negative ion mode.

#### 2.2.3. Pre-Processing and Annotation of Data

Raw data were converted to extensible markup language (mzXML) format using ProteoWizard (https://proteowizard.sourceforge.io/, accessed on 26 March 2024). Then, data in mzXML format were processed with an in-house program, which was developed using R software, version 4.2.3 (R Institute for Statistical Computing, Vienna, Austria) and based on XML Cryptographic Message Syntax (XCMS), for the detection, extraction, alignment, and integration of peaks [13]. Then, an in-house MS/MS database (BiotreeDB) was applied for metabolite annotation. The cutoff for annotation was set at 0.3.

#### 2.2.4. Screening of Differential Metabolites (DMs)

DMs were screened with cutoffs of variable importance in projection (VIP) >1 and *p* < 0.05.

### 2.3. Transcriptomics Analysis

#### 2.3.1. Extraction, Quantification, and Qualification of RNA

The total RNA of *A. sinensis* was extracted using the RNAprep Pure Plant Kit according to the manufacturer’s (Tiangen, Beijing, China) instructions. The concentration and purity of RNA were measured using a spectrophotometer (NanoDrop™ 2000; Thermo Fisher Scientific). RNA integrity was assessed using the RNA Nano 6000 Assay Kit of a Bioanalyzer (2100 series; Agilent Technologies, Carlsbad, CA, USA).

#### 2.3.2. Library Preparation for Transcriptome Sequencing

RNA (1 μg) was used as input material for the preparation of an RNA sample. Sequencing libraries were generated using the Hieff NGS Ultima Dual-mode mRNA Library Prep Kit (Yeasen Biotechnology, Shanghai, China) for Illumina (San Diego, CA, USA) following the manufacturer’s recommendations. Index codes were added to attribute sequences for each sample. Briefly, messenger (m)RNA was separated from total RNA using poly-T oligo-attached magnetic beads. First-strand complementary (c)DNA was synthesized, and the synthesis of second-strand cDNA was undertaken subsequently. Remaining overhangs were converted into blunt ends via exonuclease/polymerase activities. After adenylation of the 3′ ends of DNA fragments, the NEBNext^®^ Adaptor (New England Biolabs, Ipswich, MA, USA) with a hairpin loop structure was ligated in preparation for hybridization. Library fragments were purified with the AMPure XP system (Beckman Coulter, Brea, CA, USA). Then, 3 μL of USER™ Enzyme (New England Biolabs) was used with size-selected, adaptor-ligated cDNA at 37 °C for 15 min, followed by 5 min at 95 °C before the polymerase chain reaction (PCR). Then, PCR was undertaken with Phusion™ High-Fidelity DNA Polymerase, Universal PCR primers, and Index (X) Primer, which were all from Thermo Fisher Scientific. PCR products were purified (AMPure XP system). Library quality was assessed on the Bioanalyzer (2100 system). Libraries were sequenced on the NovaSeq™ platform (Illumina) to generate 150 bp paired-end reads according to the manufacturer’s instructions.

#### 2.3.3. Gene Expression

Gene expression in different experimental samples was studied. In general, the greater the number of transcripts of a gene that are expressed, the more reads from the gene will be obtained. Therefore, the expression of the gene can be calculated according to the count of sequenced reads in each transcript.

StringTie (https://ccb.jhu.edu/software/stringtie/, accessed on 26 March 2024) was used to standardize fragments per kilobase of transcript per million fragments mapped (FPKM) as a measure of transcript expression or gene expression [14].

#### 2.3.4. Sequencing and Transcriptomics Analysis

According to the manufacturer’s (Illumina) instructions, the library was sequenced on the NovaSeq™ platform to generate a 150 bp double-terminal sequence.

#### 2.3.5. Screening of Differentially Expressed Genes (DEGs)

Genes with significantly different expressions in different samples are called DEGs. During detection of DEGs, |log2 fold change (FC)| > 1 and false detection rate (FDR) < 0.01 were used as screening criteria. FC represents the ratio of expression between two groups of samples. FDR is calculated by *p*-value correction and indicates the significance of a difference. To facilitate comparison, the logarithmic value of FC is taken. The larger the value of |log2 FC|, the smaller the FDR, and the more obvious the difference in the sample of a gene.

### 2.4. Combined Use of Metabolomics Analysis and Transcriptomics Analysis

Normalized correlation data were imported into Excel™, version 16.0.1 (Microsoft, Redmond, MA, USA). The correlation coefficient and *p*-value were calculated using the “Spearman” algorithm of R. Calculated values were analyzed jointly.

### 2.5. Validation of Related Genes by Real-Time Reverse Transcription-Quantitative Polymerase Chain Reaction (RT-qPCR)

Seven key genes were selected from DEGs for validation by real-time RT-qPCR. With actin (ACT) as the internal reference gene, primers were designed according to the required sequence of the target gene. Primer information is shown in Table 1. RNA was extracted, and the corresponding cDNA was synthesized using a reverse transcription kit (Beijing Quanshijin Biotechnology, Beijing, China) for validation by real-time RT-qPCR.

### 2.6. Data Analysis

Excel, Origin version 2021 (OriginLab, Northampton, MA, USA), Prism 10 (GraphPad, La Jolla, CA, USA), and SPSS 25 (IBM, Armonk, NY, USA) were used for data analyses.

## 3. Results

### 3.1. Metabolomics Analysis of WA and CA

#### 3.1.1. DM Classification

**Figure 2:**
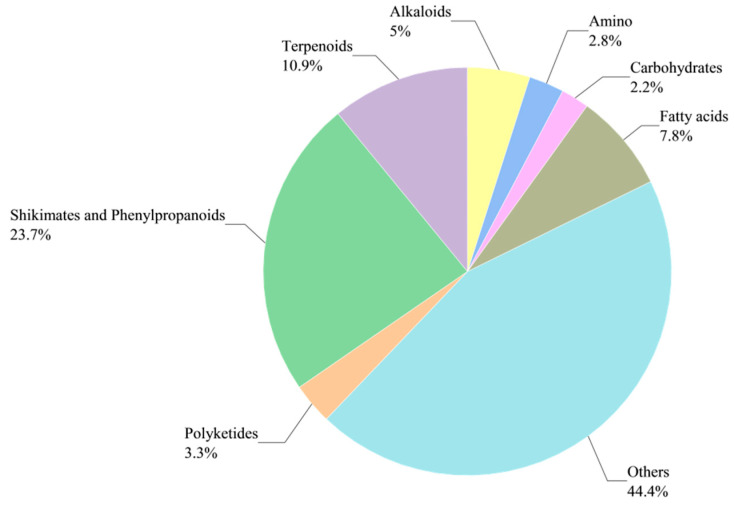
Pie plot of differential metabolite classification and proportion.

Principal component analysis (PCA) revealed that the PC1 and PC2 values for WA/CA accounted for 42.2% and 18.9%, respectively (Appendix A). The metabolic profiles of WA and CA exhibited distinct spatial separation, indicating significant differences between them. Many compounds were identified by UHPLC-MS. A pie chart can show the proportion of each part of the whole, and different parts can be compared intuitively. A pie chart was drawn for the identified compounds (Figure 2). Compounds with a high proportion were shikimates and phenylpropanoids (23.7%), terpenoids (10.9%), fatty acids (7.8%), alkaloids (5.0%), and polyketides (3.3%).

#### 3.1.2. Screening of DMs

**Figure 3:**
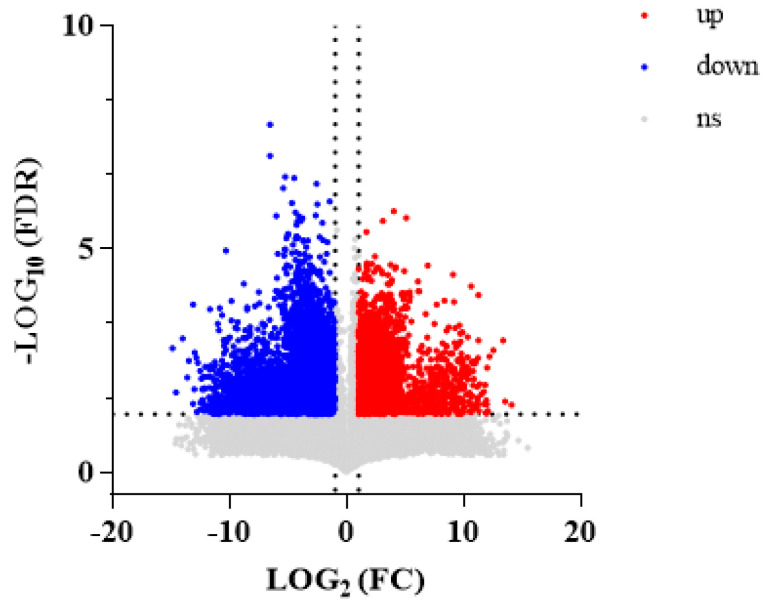
Volcanic plot of DMs between WA and CA groups (significantly UR metabolites are shown in red, significantly DR metabolites are shown in blue, and non-significantly differentiated metabolites are shown in gray; VIP > 1.0, *p*-value < 0.05).

A total of 12,580 metabolites with *p* < 0.05 were selected from 60,052 DMs. A total of 4919 DMs had upregulated expression and 7661 had downregulated expression in WA compared with CA. A volcano plot visually shows the overall distribution of DMs between the groups (Figure 3).

#### 3.1.3. Analysis of Enrichment of Pathways of DMs

**Figure 4:**
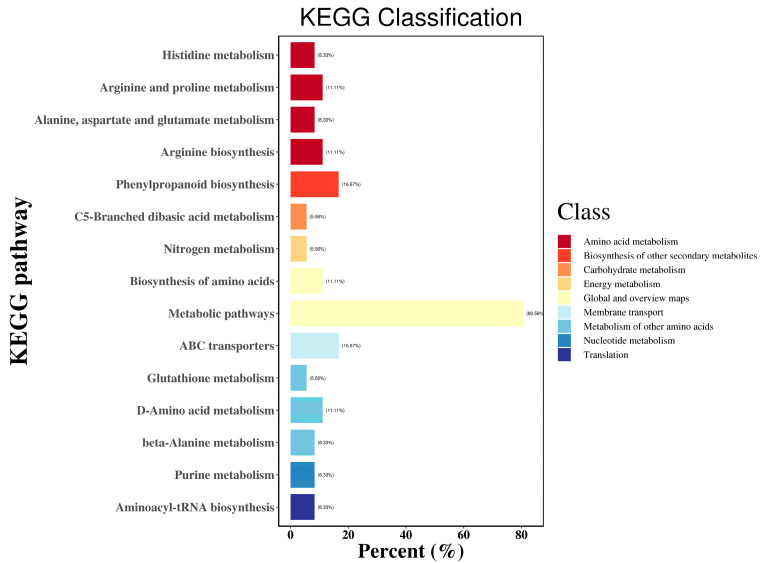
KEGG classification plot of differential metabolites.

The Kyoto Encyclopedia of Genes and Genomes (KEGG) database (www.genome.jp/kegg/, accessed on 29 March 2024) is the most commonly used database for analyses of the enrichment of pathways [15,16]. Annotation of pathways is shown in Figure 4.

**Figure 5:**
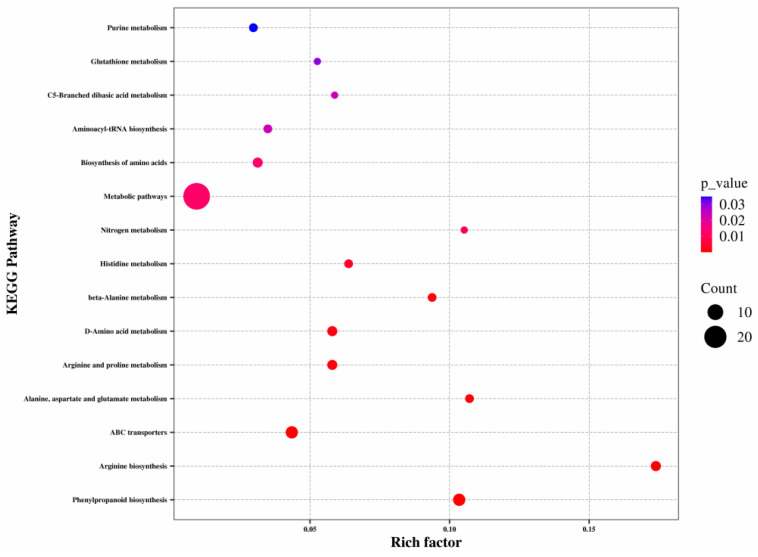
KEGG enrichment plot of differential metabolites.

More DMs were annotated under “metabolic pathways” and “phenylpropanoid biosynthesis” (Figure 5). In Figure 5, the dot representing “Metabolic pathways” is the largest, indicating that metabolic pathways were enriched with the highest number of DMs. “Arginine biosynthesis”, “Alanine, aspartate and glutamate metabolism”, and “Phenylpropanoid biosynthesis” were smaller in *p*-value and larger in Richness Factor, indicating that there were more annotated DMs in these three pathways. A combination of Figure 4 and Figure 5 helped to focus on “Metabolic pathways” and “Phenylpropanoid biosynthesis”. Cruz and colleagues reported that flavonoids produce various colors in different parts of a plant [17]. There are great differences in color between WA and CA (Figure 1). Therefore, subsequent analysis of the “Flavonoid biosynthesis pathway” was considered.

### 3.2. Transcriptomics Analysis of WA and CA

#### 3.2.1. Analysis of Gene Expression Levels

**Figure 6:**
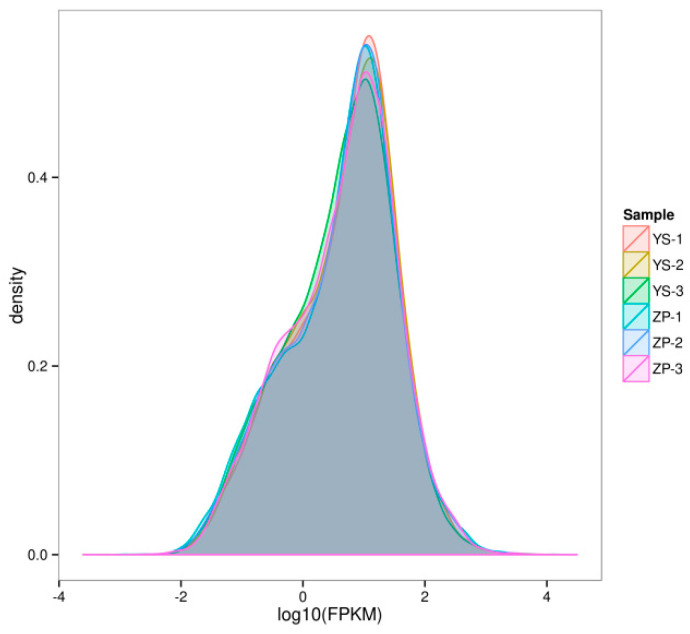
Comparison of FPKM density distribution.

Figure 6 shows the density distribution of gene expression. The distribution of the curves of six colors was essentially identical. The peak value of the curve was ~0.5, and the log10 FPKM of most genes was between 0 and 2, indicating that the consistency of gene expression of the six batches of *A. sinensis* was very high.

#### 3.2.2. DEGs

**Figure 7:**
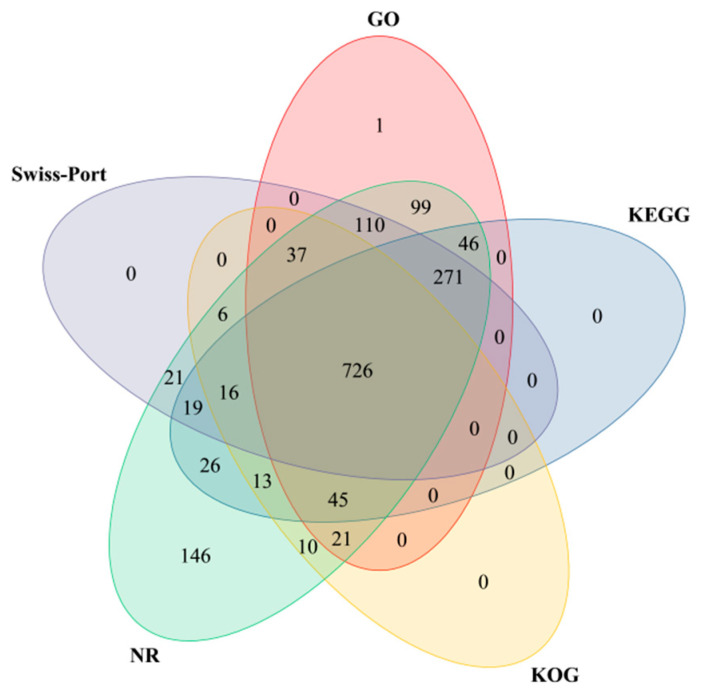
Basic annotation of the isoforms based on GO, KEGG, KOG, NR, and SwissProt databases.

A total of 21,521 DEGs detected were screened. A total of 1837 DEGs with obvious differences were screened and compared with five databases: Gene Ontology (https://geneontology.org/, accessed on 29 March 2024), KEGG, KOG (https://www.kegg.jp/, accessed on 29 March 2024), NR (http://ftp.ncbi.nih.gov/blast/db/, accessed on 29 March 2024), and SWISS-PROT (www.sib.swiss/swiss-prot/, accessed on 29 March 2024). A Venn diagram was drawn (Figure 7). A total of 1356 DEGs were annotated in the GO database, 1162 DEGs in the KEGG database, 874 DEGs in the KOG database, 1612 DEGs in the NR database, and 1206 DEGs in the SWISS-PROT database. A total of 1613 DEGs were annotated, accounting for 87.81% of the total number of DEGs. A total of 726 DEGs were annotated by the five databases, accounting for 39.52% of the total number of DEGs, indicating that only a small fraction of the DEGs can be annotated in the five databases at the same time.

**Figure 8:**
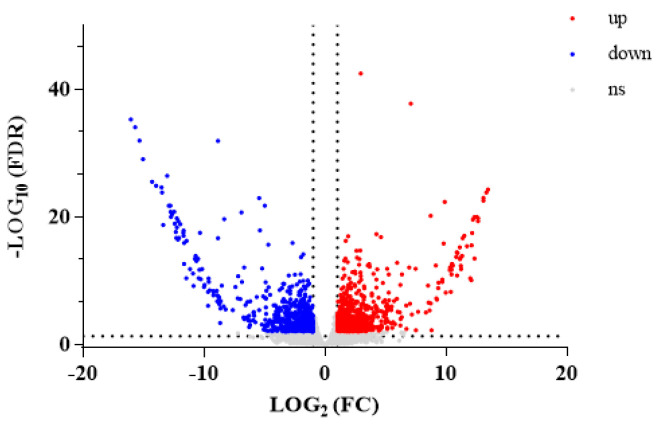
Volcano plot of DEGs.

**Figure 9:**
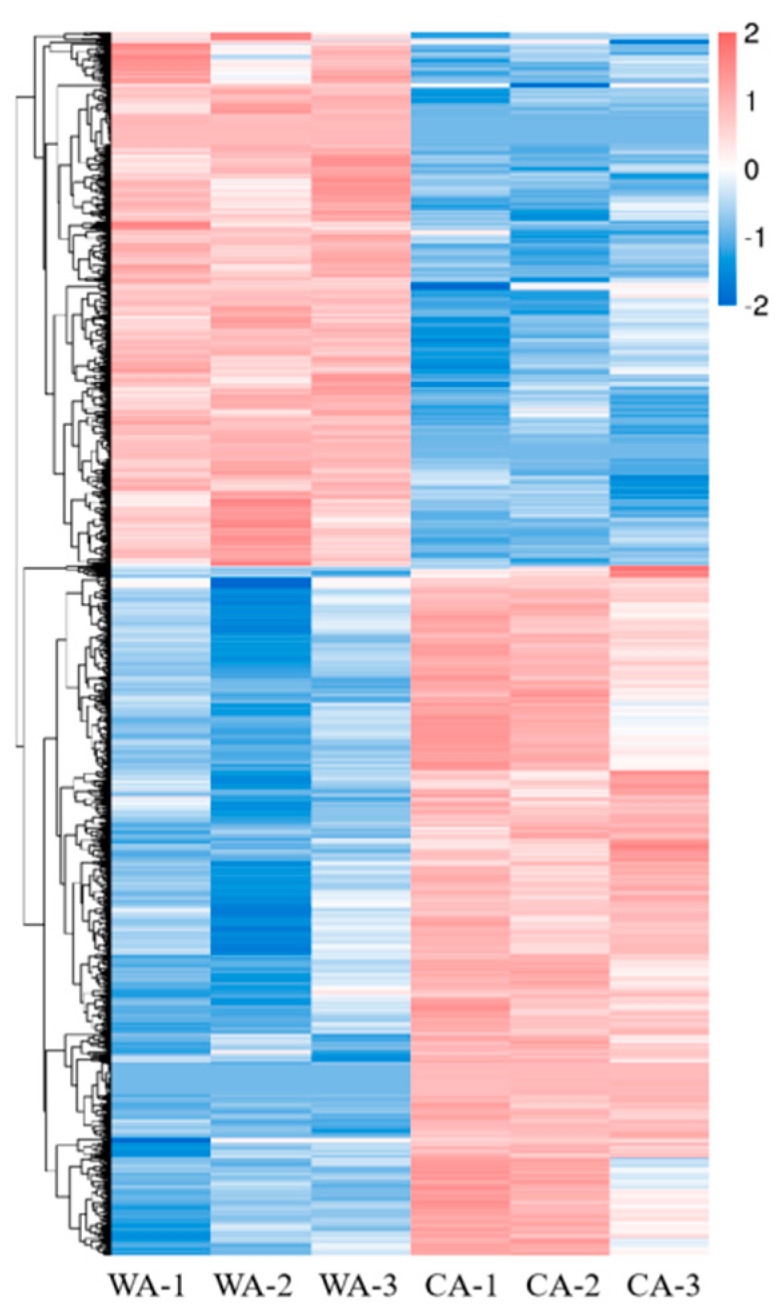
Cluster plot of DEGs.

Among 1837 DEGs, 1036 had upregulated expression and 801 had downregulated expression in WA compared with CA. A volcano plot of DEGs was drawn (Figure 8). Genes with similar expression patterns may have the same function. To visually show the differences in gene expression between WA and CA and to “mine” new functional genes, hierarchical clustering analysis was performed on all screened DEGs, and a cluster plot was drawn (Figure 9).

#### 3.2.3. Analyses of Pathway Enrichment of DEGs

**Figure 10:**
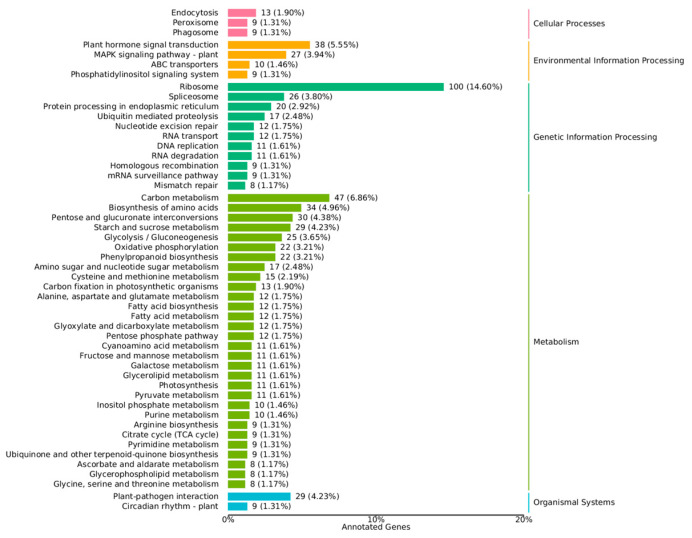
Statistical plot of KEGG enrichment analysis of DEGs.

In organisms, different genes coordinate with each other to perform biological functions. Annotation of the pathways of DEGs can aid deeper understanding of gene function. The annotation results of DEGs were classified according to the KEGG database, and a statistical plot of enrichment analysis was drawn (Figure 10). The largest number of pathways related to “Metabolism” were annotated. Among them, the pathways with more annotated genes were carbon metabolism, biosynthesis of amino acids, pentose and glucuronate interconversions, starch and sucrose metabolism, glycolysis/gluconeogenesis, oxidative phosphorylation, phenylpropanoid biosynthesis, amino sugar and nucleotide sugar metabolism, cysteine and methionine metabolism, and carbon fixation in photosynthetic organisms. The main chemical components of *A. sinensis* were volatile oils, organic acids, polysaccharides, flavonoids, and amino acids. Among them, ferulic acid and chlorogenic acid (which are organic acids) are products of phenylpropanoid biosynthesis [18,19]. Moreover, ferulic acid is one of the index components used for the quality control of *A. sinensis* in The Chinese Pharmacopoeia (2020). Therefore, the main analysis of “Phenylpropanoid biosynthesis” was carried out.

### 3.3. Combination of Metabolomics Analysis and Transcriptomics Analysis

**Figure 11:**
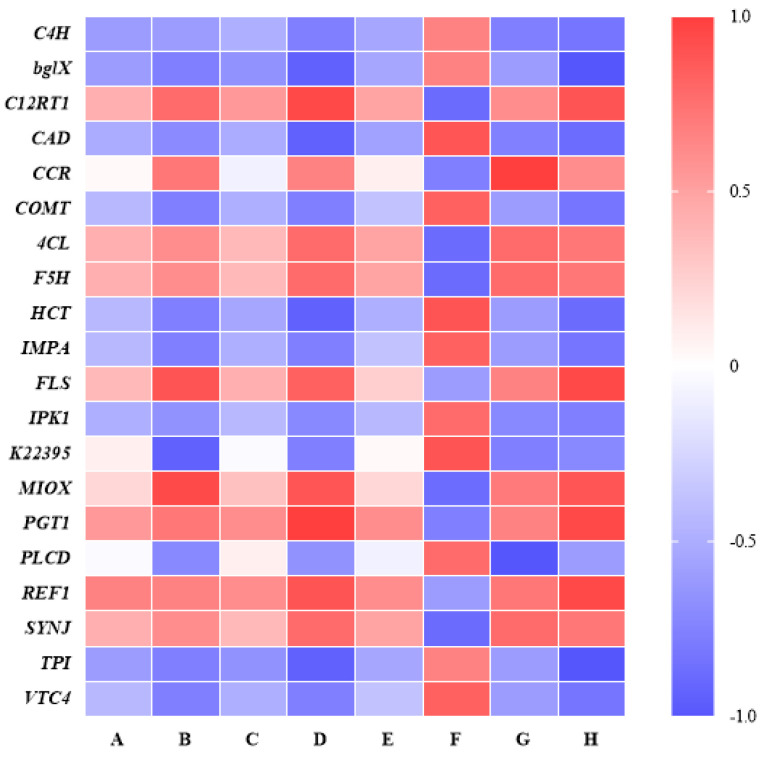
Joint analysis of heat plot (A—angelicolide, B—cinnamic acid, C—ligustilide, D—phlorizin, E—butylidenephthalide, F—2-glucosyloxy-4-methoxycinnamic acid, G—caffeoylquinic acid, H—p-coumaric acid).

We wished to compare the metabolites and genes in WA and CA more deeply. The DMs and DEGs that we wished to focus upon were analyzed using MA and TA, and a heatmap was drawn (Figure 11). Except for cinnamoyl-CoA reductase (*CCR*), cinnamyl-alcohol dehydrogenase (*K22395*), inositol oxygenase (*MIOX*), phosphatidylinositol phospholipase C, delta (*PLCD*), and other DEGs were significantly correlated with eight DMs. Therefore, a combined analysis of significantly related DMs and DEGs and their related pathways was carried out.

**Figure 12:**
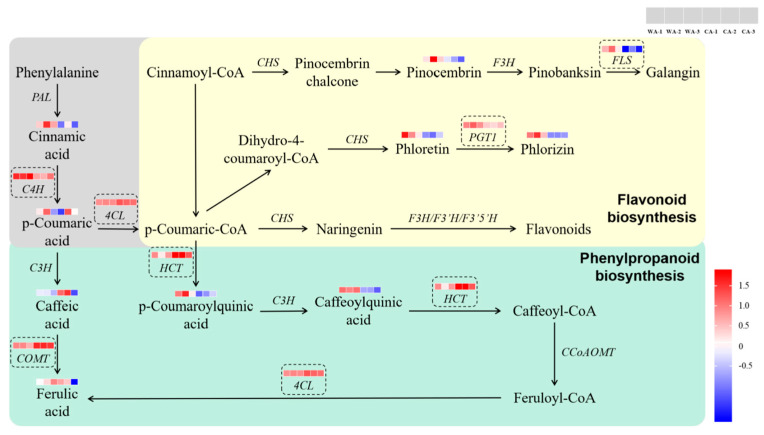
Phenylpropane biosynthesis and flavonoid biosynthesis of WA and CA (the green area is the phenylpropane biosynthesis pathway, the yellow area is the flavonoid biosynthesis pathway, and the gray area is the common part of the two pathways).

The annotation of “Phenylpropanoid biosynthesis” (map00940) and and “Flavonoid biosynthesis” (map00941) in the KEGG database was undertaken. Several DMs and DEGs that we focused upon were detected in these two pathways (Figure 12). Detection in WA vs. CA showed that in “Phenylpropanoid biosynthesis”, expression of cinnamic acid 4-hydroxylase (*C4H*) was downregulated significantly, and expressions of caffeic acid-O-methyltransferase (*COMT*), 4-coumarate-CoA ligase (*4CL*), and shikimate O-hydroxycinnamoyltransferase (*HCT*) were upregulated significantly. Expressions of the metabolites cinnamic acid, caffeic acid, *p*-coumaroylquinic acid, and caffeoylquinic acid were downregulated significantly, and those of the metabolites p-coumaric acid and ferulic acid were upregulated significantly in “Phenylpropanoid biosynthesis” (Figure 12). The previous study of our research group found that the content of ferulic acid in CA was significantly higher than that in WA [20]. It can be speculated that *C4H*, *COMT*, *4CL*, and *HCT* may be the key genes that produce significant differences in ferulic acid content between WA and CA. Phenylpropanoid biosynthesis plays an important part in the growth and development of plants. The response to adversity stress is closely related to the synthesis of many active ingredients in medicinal plants [21]. The catalytic reaction of the first three steps in the phenylpropanoid–biosynthesis pathway is considered to be the core of the entire pathway. The enzymes that play a key part are phenylalanine ammonia lyase (*PAL*), *C4H*, and *4CL* [22]. Ferulic acid is one of the products of the phenylpropanoid–biosynthesis pathway. In A. sinensis, ferulic acid is synthesized and accumulated through the COMT pathway and caffeoyl-CoA-O-methyltransferase pathway [19]. Studies have found that *4CL* is involved in the biological metabolism of ferulic acid, and *COMT* may play a key part in the biosynthesis of ferulic acid and phthalides [23,24,25]. A combination of the results of MA and TA suggested that *4CL* and *COMT* may be the key genes with significant differences in ferulic acid content between WA and CA.

Flavonoids are important chemical components in A. sinensis. Flavonoids have antibacterial, antiviral, antioxidant, and other effects, but also have important roles in the growth, development, and ecological defense of *A. sinensis* and other plants. Flavonoids affect the color of plants [26], improve resistance to pathogens [27], and improve the tolerance and resistance of a plant to abiotic stress [28,29,30,31], and so on. In terms of “Flavonoid biosynthesis”, expression of *C4H*, phlorizin synthase (*PGT1*), and flavonol synthase (*FLS*) was downregulated significantly, and *4CL* expression was upregulated significantly. Expressions of the metabolites cinnamic acid, phloretin, phlorizin, and pinocembrin were downregulated significantly, and expression of the metabolite *p*-coumaric acid was upregulated significantly (Figure 12). These results showed that *C4H*, *4CL*, *PGT1*, and *FLS* may be related to the differences in flavonoid content between WA and CA. Therefore, we speculated that these four genes were also related to the color difference between WA and CA. Studies have found that phlorizin is a common dihydrochalcone compound, which has a slightly sweet taste, and it includes antioxidant, antibacterial, antiviral, antitumor, and other biological activities [32]. The results of DM detection showed that phlorizin expression was downregulated significantly in WA compared with that in CA. These data indicated that phlorizin may lead to differences in some biological activities between WA and CA, and may also be one of the important factors leading to the difference in taste between WA and CA. Studies have reported that galangin is a flavonoid with anti-inflammatory, antibacterial, anti-aging, and anti-hypertensive effects [33]. *FLS* is located upstream of galangin, and galangin expression was downregulated significantly in WA compared with that in CA. We speculate that FLS is one of the key genes responsible for the difference between some biological activities of WA and CA.

In addition to the nine DMs in Figure 12, there are five significant DMs in “Phenylpropanoid biosynthesis” and “Flavonoid biosynthesis”: 4-hydroxystyrene, *p*-coumaryl alcohol, sinapic acid, conife rin, and sinapyl alcohol. These five DMs were significantly downregulated in WA vs. CA. *CAD* is located upstream of *p*-coumaryl alcohol and sinapic acid, and *COMT* is located upstream of sinapic acid. It can be speculated that *CAD* may regulate *p*-coumaryl alcohol and sinapic acid, and *COMT* may regulate sinapic acid.

### 3.4. Validation of Data Using Real-Time RT-qPCR

**Figure 13:**
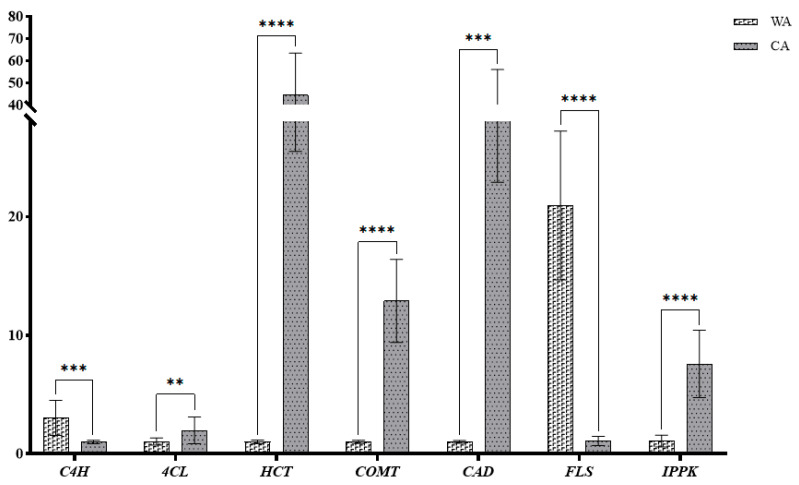
The RELs of genes involved in phenylpropane biosynthesis and flavonoid biosynthesis (** *p* < 0.05; *** *p* < 0.001; **** *p* < 0.0001).

A histogram of the relative expression of seven key genes was drawn (Figure 13). The results showed that the verification results of qRT-PCR were consistent with transcriptomic analysis. This indicated that the data from transcriptomic analysis were reliable.

## 4. Discussion

Although some references have previously studied the biosynthesis pathways and related genes of the active ingredients in *A. sinensis,* there are few omics studies based on WA and CA. In addition to several common key genes in “Phenylpropanoid biosynthesis” of *A. sinensis*, we also found that expressions of cinnamyl-alcohol dehydrogenase (*CAD*), cinnamoyl-CoA reductase (*CCR*), and coniferyl-aldehyde dehydrogenase (*REF1*) were downregulated significantly. It has been reported that both *CAD* and *CCR* are related to the biosynthesis of lignin [34,35,36,37,38], and *REF1* is involved in the biosynthesis of ferulic acid and sinapic acid [39]. Therefore, it is speculated that *CAD*, *CCR*, and *REF1* are also the key genes responsible for the differences in “Phenylpropanoid biosynthesis” between WA and CA. Relevant research has shown that chalcone synthase (*CHS*) is a key gene linking “Phenylpropanoid biosynthesis” to “Flavonoid biosynthesis”. *CHS* can catalyze the coumarin-CoA reaction to synthesize naringenin chalcone [40]. However, in this study, there was no significant difference in *CHS* between WA and CA. The expression of *CHS* is regulated at multiple levels and is not the sole factor determining the final accumulation of flavonoids. This finding suggests that the differences between WA and CA may not have reached the threshold necessary to trigger significant changes in *CHS* expression, or that any potential variations are masked by other compensatory regulatory mechanisms. Therefore, this indicates that *CHS* was not the key gene responsible for the differences in “Flavonoid biosynthesis” between WA and CA.

In addition to organic acids and flavonoids, volatile oils were the main components of *A. sinensis*. Volatile oils and ligustilide are index components used for the quality control of *A. sinensis* in The Chinese Pharmacopoeia (2020). Volatile oils are mainly composed of phthalides, terpenes, phenols, and alkanes [41]. Phthalides are important parts of volatile oils in *A. sinensis.* Fifty-five phthalides, including *Z*-ligustilide, *E*-ligustilide, 3-butylidenephthalide, senkyunolide I, senkyunolide H, senkyunolide G, senkyunolide A, and senkyunolide J, have been identified from the extracts of *A. sinensis* [42]. Therefore, it is necessary to analyze the biosynthetic pathways of phthalides. However, phthalides are unstable and readily affected by light, temperature, and other factors, resulting in diverse structures that hamper research on phthalides. Research on the biosynthetic pathways of phthalide is scarce, and the mining and functional analysis of key genes are lacking. Studies have shown that six enzymes (phospho-2-dehydro-3deoxyheptonate aldolase 2, shikimate dehydrogenase, primary amine oxidase-like, polyphenol oxidase, tyrosine decarboxylase, and shikimate O-hydroxycinnamoyl transferase) may be involved in the biosynthetic pathways of phthalides [43]. DM detection showed that the expressions of three phthalein derivatives (angelicolide, ligustilide, and butylidenephthalide) were downregulated significantly in WA compared with those in CA. These data indicate that the contents of these three phthalide derivatives in CA may be lower than those in WA. Research has shown that volatile oil extracted *from A. sinensis* exhibits various pharmacological effects, including lowering blood pressure, protecting against ischemia–reperfusion injury, bronchodilation, and anti-inflammatory actions [44]. Among these, phthalide compounds can promote the translocation of the nuclear receptor Nur77 from the nucleus to the mitochondria, interacting with tumor necrosis factor receptor-associated factor 2 (TRAF2) and sequestosome 1 (p62), thereby inducing mitophagy to exert their anti-inflammatory effects [45]. Based on this mechanism, we speculate that the significant differences in the content of phthalide compounds between WA and CA may lead to variations in their anti-inflammatory and other pharmacological activities. Follow-up omics studies on the volatile oils of *A. sinensis* can focus on the biosynthetic pathways of phthalides and reveal many related catalases, metabolic substrates, and products.

We found that the expression of inositol-pentakisphosphate 2-kinase (*IPK1*) in the “Inositol phosphate metabolism” (map00562) pathway was upregulated significantly in WA compared with that in CA. *IPK1* is very important in plant growth because it can inhibit the growth of taproots and enhance the growth of lateral roots [46]. Phytic acid (PA) is one of the main anti-nutrients in many plants. PA can limit the bioavailability of many trace elements [47]. Studies have shown that PA has a regulatory role in phosphate homeostasis in plants and that reducing the amount of PA can make plants tolerant to environmental stresses, such as osmotic stress [48]. *IPK1* is considered to be an important gene involved in PA biosynthesis [49] and catalyzes the final step in PA biosynthesis [47]. As observed from Figure 1, the number of lateral roots of CA is significantly higher than that of WA. We speculate that *IPK1* is very likely to regulate the growth of *A. sinensis* roots, and that *IPK1* expression may also be closely correlated to the bioavailability of trace elements and tolerance to environmental stresses.

Environmental factors (e.g., temperature, light, and water availability) exert a significant influence on the accumulation of secondary metabolites [26]. After long-term artificial tending (e.g., artificial irrigation, fertilization, and pest control), the cultivation tolerance of *A. sinensis* is weakened by drought, cold, high temperature, salt, pests, and diseases [50]. We documented significant differences in the expression of inositol-phosphate phosphatase (*VTC4*) and inositol 3-alpha-galactosyltransferase (*GOLS*) between WA and CA. Studies have shown that VTC4 has good tolerance to cold stress [51], and *GOLS* has good tolerance to drought, high temperature, and cold stress [52,53]. We speculate that these two key genes are important determinants of stress tolerance in WA.

## 5. Conclusions

A combination of MA and TA was used to identify some DMs and DEGs that may lead to differences in phenylpropanoid biosynthesis and flavonoid biosynthesis between WA and CA. Network reconstruction of DEG–DM interactions highlighted that differential expression of pivotal biosynthetic genes—*C4H*, *COMT*, *4CL*, *HCT*, and *PGT1*—modulates the accumulation of phenolic acids and flavonoids, including ferulic acid, psoralen, isopsoralen, and pinosylvin, thereby contributing to the phenotypic divergence in root coloration between the two germplasms. In addition, the altered expression of stress-responsive genes (*IPK1*, *VTC4*, and *GOLS*) offers mechanistic insights into potential adaptive advantages and provides a theoretical framework for germplasm improvement and stress-resilience breeding. These findings not only supply a molecular basis for enhancing the quality of cultivated A. sinensis but also establish a foundation for more comprehensive comparative studies of wild and cultivated populations.

## Figures and Tables

**Table 1:** Primer sequence of candidate genes used for qRT-PCR validation.

Graph 5.	Primer Sequences (5′—3′)
*ACT*	Forward: TGGTATTGTGCTGGATTCTGGT
	Reverse: TGAGATCACCACCAGCAAGG
*C4H*	Forward: GCTCACTGGGAAAACCCTGA
	Reverse: ATGCAAGGATGATTCCGGGG
*4CL*	Forward: GTTGTCAGATCCCCCGACAG
	Reverse: TCCCTGAAGCTGACTTTGGC
*HCT*	Forward: TCAGTTTGGGATGAGGTGCC
	Reverse: TTTGTGTGAAACGCTGGCTG
*COMT*	Forward: AGCTTCTCATGTCGAACCCC
	Reverse: TCCAGCAGTAGAGCCACTCA
*CAD*	Forward: ACAGGAGCAACGCTAGACAG
	Reverse: AAGGACCGGCAGTCTTGA
*FLS*	Forward: AGTGTGGCGTGAGTTCTTCC
	Reverse: ACGGCTGCCATAACCTTCAT
*IPPK*	Forward: CTAAGCGTCCTTCATGGCGA
	Reverse: TCCGCACTTGGGCTTTATCT

**Figure A1:**
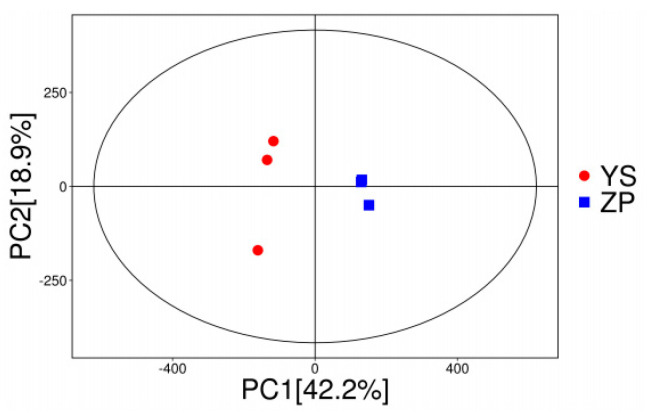
Score scatter plot for PCA.

## Data Availability

The datasets are publicly available at NCBI with PRJNA1110837.

## Abbreviations

The following abbreviations are used in this manuscript:

TCM	Traditional Chinese medicine
WA	Wild *Angelica sinensis*
CA	Cultivated *Angelica sinensis*
MA	Metabolomics analysis
TA	Transcriptomics analysis
RT-qPC	Real-time reverse transcription-quantitative polymerase chain reaction
DMs	Differential metabolites
DEGs	Differentially expressed genes
UHPLC	Ultrahigh-performance liquid chromatography
PCA	Principal component analysis
VIP	Variable importance projection
PCR	polymerase chain reaction
FPKM	Fragments per kilobase of transcript per million fragments mapped
KEGG	Kyoto Encyclopedia of Genes and Genomes
*C4H*	4-hydroxylase
*HCT*	Shikimate o-hydroxycinnamoyltransferase
*4CL*	4-coumarate-CoA ligase
*COMT*	Caffeic acid-o-methyltransferase
*CAD*	Cinnamyl-alcohol dehydrogenase
*FLS*	Flavonol synthase
*CCR*	Cinnamoyl-coa reductase
*K22395*	Cinnamyl-alcohol dehydrogenase
*MIOX*	Inositol oxygenase
*PLCD*	Phosphatidylinositol phospholipase c delta
*PAL*	Phenylalanine ammonia lyase
*PGT1*	Phlorizin synthase
*REF1*	Coniferyl-aldehyde dehydrogenase
*CHS*	Chalcone synthase
*IPK1*	Inositol-pentakisphosphate 2-kinase
*VTC4*	Inositol-phosphate phosphatase
*GOLS*	3-alpha-galactosyltransferase
*ACT*	Actin
TRAF	factor receptor-associated factor 2
p62	sequestosome 1
